# Healthcare use and costs among individuals receiving mental health services for depression within primary care in Nepal

**DOI:** 10.1186/s12913-022-08969-1

**Published:** 2022-12-30

**Authors:** Luke R. Aldridge, Emily C. Garman, Bryan Patenaude, Judith K. Bass, Mark J. D. Jordans, Nagendra P. Luitel

**Affiliations:** 1 Johns Hopkins University, Baltimore, USA; 2 University of Cape Town, Cape Town, South Africa; 3 King’s College London, London, UK; 4 Transcultural Psychosocial Organization Nepal, Kathmandu, Nepal

**Keywords:** Mental healthcare, Depression, Integration, Service costs, Low- and middle-income countries

## Abstract

**Background:**

Integrating mental health services into primary care is a key strategy for reducing the mental healthcare treatment gap in low- and middle-income countries. We examined healthcare use and costs over time among individuals with depression and subclinical depressive symptoms in Chitwan, Nepal to understand the impact of integrated care on individual and health system resources.

**Methods:**

Individuals diagnosed with depression at ten primary care facilities were randomized to receive a package of integrated care based on the Mental Health Gap Action Programme (treatment group; TG) or this package plus individual psychotherapy (TG + P); individuals with subclinical depressive symptoms received primary care as usual (UC). Primary outcomes were changes in use and health system costs of outpatient healthcare at 3- and 12-month follow up. Secondary outcomes examined use and costs by type. We used Poisson and log-linear models for use and costs, respectively, with an interaction term between time point and study group, and with TG as reference.

**Results:**

The study included 192 primary care service users (TG = 60, TG + *P* = 60, UC = 72; 86% female, 24% formally employed, mean age 41.1). At baseline, outpatient visits were similar (− 11%, *p* = 0.51) among TG + P and lower (− 35%, *p* = 0.01) among UC compared to TG. Visits increased 2.30 times (*p* < 0.001) at 3 months among TG, with a 50% greater increase (*p* = 0.03) among TG + P, before returning to baseline levels among all groups at 12 months. Comparing TG + P to TG, costs were similar at baseline (− 1%, *p* = 0.97) and cost changes did not significantly differ at three (− 16%, *p* = 0.67) or 12 months (− 45%, *p* = 0.13). Costs among UC were 54% lower than TG at baseline (*p* = 0.005), with no significant differences in cost changes over follow up. Post hoc analysis indicated individuals not receiving psychotherapy used less frequent, more costly healthcare.

**Conclusion:**

Delivering psychotherapy within integrated services for depression resulted in greater healthcare use without significantly greater costs to the health system or individual. Previous research in Chitwan demonstrated psychotherapy determined treatment effectiveness for people with depression. While additional research is needed into service implementation costs, our findings provide further evidence supporting the inclusion of psychotherapy within mental healthcare integration in Nepal and similar contexts.

**Supplementary Information:**

The online version contains supplementary material available at 10.1186/s12913-022-08969-1.

## Introduction

One in 27 people with depression, the second largest contributor to global disability, receives minimally adequate care mental health services in low- and middle-income countries (LMIC) [1, 2]. Integrating evidence-based mental health services into primary care and other existing health service delivery platforms has been recognized and implemented as a leading strategy for reducing the gap between burden and available mental healthcare in LMIC [3–7].

Addressing the mental health treatment gap is integral to achieving goals for sustainable development set by the United Nations [8], particularly that of universal health coverage [9, 10]. Universal health coverage is defined by two dimensions: that all people should receive needed healthcare (i.e., service coverage), and those who do receive care should not suffer financial hardship as a result (i.e., financial protection) [11]. The latter presents a challenge to health delivery systems primarily structured on individual out-of-pocket (OOP) expenditure to finance service provision, which represents a regressive form of health system financing [12]. Costs to the health system are also of paramount importance to healthcare administrators and policy makers when considering which health innovations to scale up and include as part of essential healthcare packages [13]. As such, researchers must examine the financial and economic impact to individuals and health systems when implementing and scaling up novel approaches to mental health services.

Some evidence from high-income countries indicates treatment for depression can improve economic outcomes, including reduced healthcare costs, though the evidence is not conclusive [14]. Research is sparser on the effects of services for depression in LMIC. A recent meta-analysis found an unconditional average effect size of 0.22 standard deviations for mental health interventions across all economic outcomes, with the smallest effect sizes observed in low-income countries [15]. Longitudinal evidence is also needed to more adequately understand the effects of services for depression on healthcare costs, particularly within LMIC [16].

Depression and other mental disorders are associated with increased healthcare costs to service users and health systems in both high- and low-resource settings [17–20]. A 2020 meta-analysis found adults with depression have approximately 160% greater costs than the general public, primarily resulting from direct healthcare costs and lost productivity [19]. In Ethiopia, households of individuals with depression are more likely to experience catastrophic OOP expenditure and impoverishment resulting from increased healthcare use [17]. Repeat household surveys indicated depression severity is associated with both increased healthcare use and OOP expenditure among community members in Chitwan District, Nepal, where the present study took place [21]. In South Asia, mental health problems are often expressed through physical symptoms, such as head and body aches, numbness, weakness, and exhaustion [22–24]. Moreover, depression is linked to diabetes, hypertension, cardiovascular disease, and other co-occurring physical health conditions [25]. Therefore, health service researchers have typically included healthcare costs and use for both physical and mental health concerns when working South Asian contexts, such as in the repeat household surveys above [21].

The Programme for Improving Mental Health (PRIME) was one of the largest research initiatives to implement an integrated task sharing approach to expand access to mental healthcare in LMIC [26]. PRIME implemented a multi-level district mental healthcare plan in five countries – Ethiopia, India, Nepal, South Africa, and Uganda – where, at the health systems level, mental health services were delivered by non-specialist health workers within primary care according to guidelines from the World Health Organization (WHO) Mental Health Gap Action Programme (mhGAP) [26–28]. In Nepal, where roughly 5% of people with depression had access to mental health services prior to the study [29], researchers also examined the effectiveness of including individual psychotherapy within mhGAP-based mental health services for people with depression and alcohol use disorder [30]. They found adding individual psychotherapy to a standard package of implemented mhGAP services (i.e. pharmacological and basic psychosocial services) provided by primary care workers led to larger reductions in clinical symptoms and functional impairment for those with depression, though they observed no additional benefit for people with alcohol use disorder [31].

The present study examined trends in healthcare use and costs among two groups of people with depression: those who received a standard package of care and those who received this package plus individual psychotherapy as part of mhGAP-based treatment for depression. We also studied a third group of people with subclinical depressive symptoms who received usual care (UC), which represents an approximate counterfactual of service use and costs when integrated mental health services are not provided. We examined trends in healthcare use for mental and physical health, separately and combined, to understand what drives healthcare costs. Our methods build on previous research by Chisholm et al. [32] studying health service costs and their association with functional impairment among PRIME participants in all five country sites. We extend this research by comparing trends over time and across the three study groups for depression in PRIME Nepal and by also examining healthcare use. Oure goal was to provide insight into the individual and health system impacts of including individual psychotherapy within integrated packages of care. These insights can improve our understanding of how integrated mental services affect other healthcare use, ensure those who receive expanded care do not experience undue financial burden, and ultimately inform efforts to integrate and scale up mental services within LMIC.

## Methods

### Setting

PRIME researchers, ministry officials, and healthcare workers implemented a district mental healthcare plan in Chitwan, Nepal from 2014 to 2016 [26, 33, 34]. Research prior to program implementation found that 5% of a representative sample of the district’s total population – approximately 580,000 residents at the time of study [35] – screened positive for depression [36]. However, only 8% of those who screened positive in the district had received treatment for depression in the past 12 months and less than 2% had sought treatment for depression within primary care [36]. Mental health services in Nepal prior to PRIME were largely restricted to a few government and private hospitals in major cities, though some services were also available at the district hospital and medical colleges operating in Chitwan [36].

The PRIME district mental healthcare plan implemented intervention packages at the community and health facility from 2014 to 2016 [34]. At the health facility level, health workers at ten primary care facilities provided services for depression and three other priority disorders – alcohol use disorder, psychosis, and epilepsy – according to clinical decision-making guidelines in the mhGAP Intervention Guide [38]. The present study examined healthcare use and costs among individuals receiving care for depression, as well as those with subclinical depressive symptoms, at the ten health facilities during the implementation phase. PRIME moved into program scale-up in 2016 and, since then, integrated care based on PRIME has been scaled to all 46 primary care facilities in Chitwan [39]. The Ministry of Health is also scaling up an adapted package of mental health services in select primary care facilities in other districts based on results and protocols from the PRIME model, though the Ministry's protocols do not include individual psychotherapy or plans to train auxiliary health workers as of 2020 [39].

### Study design and recruitment

PRIME research assistants screened eligible service users at the ten participating primary care facilities for depression during routine healthcare visits using an adapted and validated version of the nine-item Patient Health Questionnaire (PHQ-9) [40, 41]. Eligible criteria required participants to be 16 years of age (i.e., the age of majority in Nepal) or older, reside in Chitwan, be fluent in the local language, willing and able to provide informed consent, and not already receiving mental health services for depression.

Individuals who screened positive for depression, as indicated by a PHQ-9 score greater or equal to 10, were further assessed by a trained medical officer or trained primary care worker using the mhGAP protocol. Participants who met the criteria in the mhGAP Intervention Guide [38] for depression during diagnostic interviews were invited to enroll in a randomized trial comparing two packages of care, while a few trial participants were assessed directly by a healthcare worker without prior PHQ-9 screening. The two packages of care were a standard treatment group (TG) consisting of psychoeducation, emotional support, and antidepressant medication when indicated, versus these same services plus individual psychotherapy (TG + P), with both packages delivered according to mhGAP guidelines [30]. A total of 120 participants were diagnosed with depression and allocated to TG (*n* = 60) or TG + P (n = 60). The psychotherapy provided in TG + P was an adapted version of the Healthy Activity Programme [42], an evidence-based behavioral activation treatment developed in India to be delivered over six to eight weekly sessions. Psychotherapy sessions were delivered by psychosocial counsellors in the community [39]. Those who did not meet depression diagnostic criteria but who screened positive on the PHQ-9, i.e., those who reported subclinical depressive symptoms, were recruited into a separate comparison cohort that received primary care as usual (UC) (Supplementary Fig. S1). In summary, two treatment groups (TG and TG + P) and one comparison group (UC) were included in the present study. Further information on the PRIME study protocol [28], pilot testing [34], program process evaluation [43] and impact [31, 33, 44] are available elsewhere.

### Measures

Participants in all three groups completed a battery of questionnaires at baseline and 3- and 12-month follow up [28], which included measures of sociodemographic information at baseline and depressive symptoms [41] and functional impairment [45] at each time point. Research assistants also administered items from the Client Socio-Demographic and Service Receipt Inventory (CSRI) [46], which has been adapted for use in South Asia [47], to measure participant healthcare use and costs at all time points. The CSRI is a brief self-report measure in which participants are asked about recent visits to various formal and informal healthcare providers. In the outpatient healthcare section, respondents are asked about the date, cause, duration, and cost of each outpatient visit in the past three months to a range of healthcare providers, including biomedical, traditional, and complementary healthcare providers. PRIME researchers used a three-month time period for outpatient healthcare use to accommodate service user recall; there is evidence respondents underreport outpatient healthcare use beyond this period [48, 49]. The CSRI also include items where respondents as asked to report the type, dose, and frequency of medication prescribed during each healthcare visit.

### Outcomes

We examined changes in multiple categories of healthcare use and costs over time across study groups. Our primary outcome for healthcare use was defined as all outpatient healthcare visits to any provider in the prior three months, including those in the formal and informal healthcare sectors. We include visits to providers in both sectors given the role traditional and religious healers play within help-seeking for mental health concerns [50]. As secondary analyses, we examined outpatient visits for mental and physical health separately, categorizing each visit by the primary presenting health concern.

Our primary cost outcome was total cost to the health system for all outpatient visits in the prior three months. We adopted a societal perspective when estimating costs [51], which combined service user economic costs with costs to government for providing public healthcare services and psychotropic medications. Estimating service user economic costs required valuing individual opportunity costs for healthcare use. To do so, we proxied opportunity costs as the monetary value of time spent utilizing healthcare, derived by multiplying the average wage per minute of all participants by participant-reported time spent traveling to and from, waiting for, and accessing health services. Public sector costs for service provision were estimated by combining units of healthcare use – reported via CSRI – with published unit costs from WHO [32, 52, 53]. Secondary cost analyses examined three specific categories of costs: a) health system cost for mental health, b) total individual OOP expenditure, and c) individual OOP expenditure on mental health. Individual OOP expenditures were self-reported using the CSRI and include consultation and transportation costs to the individual. All outcomes are reported for the previous three months and focus on outpatient healthcare. Costs are reported in 2020 international dollars.

Cost categorizations above reflect best practices in defining healthcare costs [54] and generally align with the health system costing approach of Chisholm et al. [32] in a broader study examining healthcare costs of service user cohorts across all five PRIME country sites. Our methods differ from Chisholm et al. [32] in a few notable ways. First, we assign costs for psychotropic medications to the healthcare provider under the health system perspective, rather than to the service user as OOP expenditure, since most psychotropic medications were provided by PRIME at no cost to the service user. Medication costs are estimated in both studies by multiplying self-reported prescribed quantities to published WHO unit costs [32, 52, 53]. Second, our study focuses on outpatient healthcare, excluding inpatient healthcare from our analyses. Inpatient admissions were rare among our sample (< 2%) and their inclusion would have resulted in highly skewed cost estimates among small number of study participants as outliers. Third, we adjusted all costs reported by service users in Nepalese rupees to the year 2020, the most recent year for which the purchasing power parity conversion factor was available from the World Bank [55] at time of writing, and converted to international dollars using purchasing power parity (100 Nepalese rupees = 33.5 international dollars; 1 United States dollar = 3.53 international dollars in Nepal). We report results in international dollars, rather than in 2014–2016 Nepalese rupees, to promote external generalizability of healthcare costs [56]. Last, we define mental healthcare as healthcare visits at any provider where the primary reason for seeking care was for mental health, rather than restricting to only care received at mental health facilities [32]. Our definition of mental healthcare aligns with current efforts to expand access to mental healthcare and extends the definition to cover care received at home by public sector health works (e.g., community health workers, social workers), including home-based follow up and community counseling provided under PRIME.

### Analysis

We analyzed changes over time for each outcome using regression models with an interaction term between study group and each follow up time point, using TG as reference. We chose TG as reference to promote the comparability of results among treatment groups (i.e., TG and TG + P). Moreover, UC would have been an imperfect reference because nonrandom participant allocation to UC limits the validity of comparisons to this group. Outpatient healthcare visits were modeled using Poisson regression for right-skewed count data, which produced estimates of incidence rates (i.e., the number of expected healthcare visits over the three-month time period) and incidence rate ratios. Healthcare costs were modeled using log-linear regression to account for non-normal distributions and reduce model sensitivity to outliers. Coefficients estimated using log-linear models indicate the percentage change – or more specifically, 100*(e^β^–1)% – in expenditure for each unit change in explanatory variables [57], which in the present study are differences between study groups and time points. Interaction coefficients in the models represent the difference in difference between study groups over time. Outliers were considered to be observations with cost data more than three standard deviations above log-transformed values and were removed prior to analysis. Missing data were handled in a two-step process. First, where individual items were missing within the outpatient healthcare or medication sections, mean values were imputed in line with methods from Chisholm et al. [32]. We then used multiple imputation where entire sections were missing due to loss to follow up or nonresponse [58]. All analyses were conducted in Stata version 15.1.

## Results

### Sample

One hundred ninety-one individuals were enrolled in either UC (*n* = 72) or the treatment groups TG (*n* = 60) and TG + P (n = 60) (Table 1;Supplementary Fig. S1). The majority of participants were female (TG = 88%, TG + *P* = 82%, UC = 87%), not employed (TG = 72%, TG + *P* = 74%, UC = 77%), and married or partnered (TG = 88%, TG + *P* = 75%, UC = 89%). Average age across all groups at baseline was 40.5 (standard deviation (SD) = 13.9; TG = 43.5 [SD = 13.4]; TG + *P* = 39.0 [SD = 14.1]; UC = 39.2 [SD = 14.1]). Forty participants (21%) were lost to follow up due to moving away from the study area, refusal to continue participation, or other reasons (Supplementary Fig. S1). Three participants (2%) reported implausibly high healthcare costs (i.e., more than three standard deviations greater than the average of log-transformed values) and were remove prior to analysis. Missing data for those lost to follow up were multiply imputed and included in analysis. Preliminary analysis indicated there were no significant differences in depression symptom scores across groups at baseline (*p* = 0.40), however average functional impairment scores were significantly lower at baseline among UC compared to treatment groups (difference = 6.84, 95% confidence interval (CI); 2.00 to 11.67; *p* = 0.006).

**Table 1:** Sociodemographic characteristics of study participants

	Usual Care	TG	TG + P
	(*n* = 71)	(*n* = 60)	(*n* = 60)
Female	62 (87%)	53 (88%)	49 (82%)
Age of participant, mean (SD)	39.2 (14.1)	43.5 (13.4)	39.0 (14.1)
Education level			
Uneducated/illiterate	17 (24%)	22 (37%)	14 (23%)
Less than primary	20 (28%)	21 (35%)	14 (23%)
Primary school and above	34 (48%)	17 (28%)	32 (53%)
Marital status			
Single	6 (9%)	1 (2%)	7 (12%)
Has a partner	63 (89%)	53 (88%)	45 (75%)
Divorced/widowed	2 (3%)	6 (10%)	8 (13%)
Employed	15 (23%)	17 (28%)	15 (26%)
Religion			
Hindu	57 (80%)	51 (85%)	51 (85%)
Buddhist	7 (10%)	8 (13%)	4 (7%)
Christian	7 (10%)	1 (2%)	5 (8%)
Caste			
Brahmin/Chhetri	20 (28%)	21 (35%)	27 (45%)
Janajati	15 (21%)	21 (35%)	14 (23%)
Dalit	32 (45%)	15 (25%)	14 (23%)
Others	4 (6%)	3 (5%)	5 (8%)

TG Treatment Group; TG + P Treatment Group plus Psychotherapy; SD standard deviation.

### Healthcare use

#### All outpatient care

**Fig. 1:**
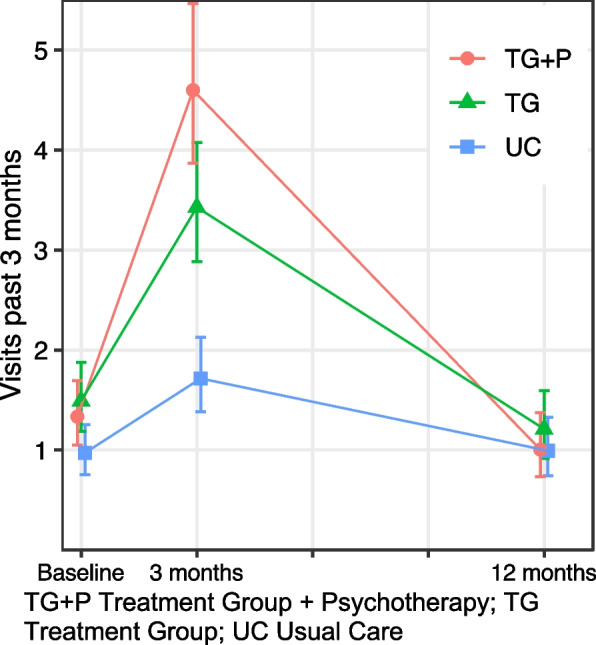
All outpatient healthcare use

Baseline healthcare use was similar among the two treatment groups and 35% lower (95% confidence interval (CI): − 54, − 8%) lower among UC (Table 2, Fig. 1). As expected, individuals receiving TG + P had the highest use at 3-month follow up of any group, a 50% (95% CI: 4, 116%) greater increase in visits from baseline relative to TG. Those in the UC group had a small increase in visits at 3 months, though remained significantly lower than those in the treatment groups (TG incidence rate (IR) = 3.43, 95% CI: 2.88, 4.07; TG + *P* = 4.60, 95% CI: 3.87, 5.47; UC IR = 1.72, 95% CI: 1.38, 2.13). Healthcare use returned to baseline levels at 12 months for all groups, with no significant differences in use between groups.

**Table 2:** ﻿Outpatient healthcare use in the past 3 months

	All health		Mental health		Physical health	
	IRR	95% CI	IRR	95% CI	IRR	95% CI
**Group**						
TG	ref.		ref.		ref.	
UC	0.65	(0.46, 0.92)	0.12	(0.04, 0.36)	0.90	(0.60, 1.34)
TG + P	0.89	(0.64, 1.24)	0.63	(0.33, 1.21)	1.02	(0.68, 1.53)
**Timepoint**						
Baseline						
3 months	2.30	(1.78, 2.96)	4.83	(3.19, 7.30)	1.10	(0.76, 1.59)
12 months	0.81	(0.58, 1.13)	1.08	(0.61, 1.91)	0.69	(0.46, 1.04)
**Group by timepoint**						
UC at 3 months	0.77	(0.52, 1.14)	0.43	(0.12, 1.57)	1.55	(0.94, 2.56)
UC at 12 months	1.26	(0.78, 2.05)	1.06	(0.24, 4.71)	1.46	(0.83, 2.56)
TG + P at 3 months	1.50	(1.04, 2.16)	2.70	(1.42, 5.12)	0.67	(0.39, 1.15)
TG + P at 12 months	0.93	(0.57, 1.51)	1.27	(0.54, 2.99)	0.85	(0.46, 1.55)

CI confidence interval; IRR incident rate ratio; TG Treatment Group; TG + P Treatment Group plus Psychotherapy; UC Usual Care.

#### Mental healthcare

**Fig. 2:**
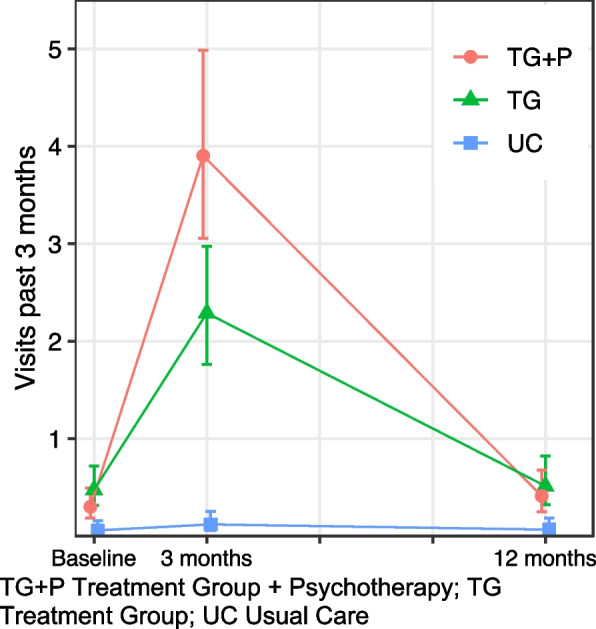
Outpatient mental healthcare use

We found similar patterns when examining mental healthcare use, i.e., outpatient healthcare visits to any provider for mental health in the past three months (Table 2, Fig. 2). Both models were inverse "V" shaped among treatment groups, suggesting increases in all healthcare use among these groups were largely explained by mental healthcare. Incidence rates were similar among the treatment groups at baseline, increased to varying degrees at 3-month follow up, with the largest increase among the TG + P, before returning to baseline levels at 12-month follow up. Mental healthcare visits were 4.83 times greater (95% CI: 3.19, 7.30) at three months compared to baseline among TG; this difference in follow up visits was 2.70 times (95% CI:1.42, 5.12) greater among TG + P, likely explained by additional visits as part of individual psychotherapy. There were roughly no healthcare visits for mental health among UC at all time points.

#### Physical healthcare

**Fig. 3:**
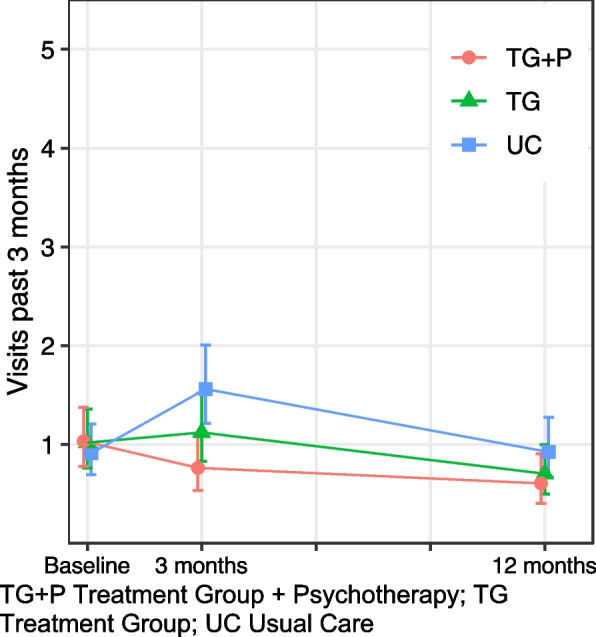
Outpatient healthcare use

A different pattern emerged when examining healthcare use for physical health. Incidence rates for physical healthcare visits are relatively low at all timepoints compared to all use and mental healthcare use, and there were no significant differences in study group, follow up, or interactions between the two. Again, we found similar rates of use among the three groups at baseline; all groups reported roughly one physical healthcare visit in the past 3 months (Table 2, Fig. 3). However, there were nonsignificant trends downwards over time for those receiving TG + P relative to TG, with a 33% (95% CI: − 61, 15%) greater decrease in visits at 3 months and 15% (95% CI: − 54, 55%) greater decrease at 12 months, i.e., physical healthcare use was slightly lower over time among TG + P compared to TG, though these differences were nonsignificant (Fig. 3). Those in the UC group had 55% (95% CI: − 6, 156%) and 46% (95% CI: − 17, 156%) greater increases in use at 3 and 12 months, respectively, compared to TG. These represent relatively moderate, but small absolute, increases in physical healthcare visits, with no significant differences between groups over time.

### Healthcare costs

#### Health system costs for all healthcare

**Fig. 4:**
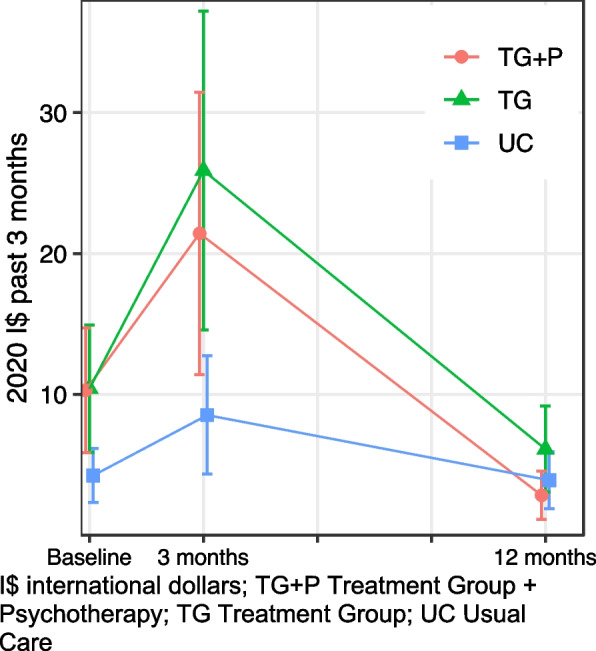
All healthcare costs

Patterns of total health system costs were similar to those of all healthcare use, though differences between treatment groups were nonsignificant (Fig. 4, Table 3). As described above, health system costs from the societal perspective are presented in 2020 international dollars and include individual OOP expenditure, time costs to individual, and costs to government for providing public healthcare services and psychotropic medications [51]. At baseline, the UC group had 54% (95% CI: − 132, − 24%) lower costs, driven by lower baseline healthcare use and approximately zero costs incurred by the government for providing psychotropic medications. Healthcare costs were greater on average among TG compared to TG + P at 3-month follow up, though the difference was not statistically significant; there was a 16% lower increase (95% CI: − 95, 6%) in costs from baseline to 3 months for TG + P compared to those for TG. Post hoc analysis indicated increases in healthcare costs among TG relative to TG + P were driven by higher individual OOP expenditure stemming from higher physical healthcare use and more care received from traditional healers and pharmacists, both of which charged greater consultation fees on average than publicly provided care at local health centers.

**Table 3:** Outpatient healthcare costs in the past 3 months

	Individual OOP expenditure				Health system costs			
	All		Mental Health		All		Mental Health	
	e^β^-1	95% CI	e^β^-1	95% CI	e^β^-1	95% CI	e^β^-1	95% CI
Group								
TG	ref.		ref.		ref.		ref.	
UC	0.04	(−0.43, 0.92)	−0.17	(− 0.38, 0.10)	− 0.54	(−1.32, − 0.24)	−0.78	(−1.84, − 1.15)
TG + P	0.17	(−0.37, 1.20)	− 0.07	(− 0.31, 0.24)	−0.01	(− 0.57, 0.55)	−0.22	(− 0.61, 0.11)
Timepoint								
Baseline	ref.		ref.		ref.		ref.	
3 months	0.92	(0.06, 2.46)	0.15	(−0.13, 0.52)	1.36	(0.32, 1.40)	0.95	(0.32, 1.01)
12 months	0.27	(−0.30, 1.33)	0.14	(−0.14, 0.51)	− 0.38	(−1.02, 0.07)	− 0.56	(− 1.17, − 0.47)
Group by timepoint								
UC at 3 months	− 0.48	(− 0.77, 0.18)	− 0.14	(− 0.41, 0.25)	− 0.23	(− 1.02, 0.50)	− 0.46	(− 1.1, − 0.12)
UC at 12 months	− 0.31	(− 0.71, 0.63)	− 0.16	(− 0.43, 0.23)	0.50	(− 0.34, 1.16)	1.34	(0.37, 1.33)
TG + P at 3 months	− 0.60	(− 0.83, − 0.06)	− 0.18	(− 0.45, 0.20)	−0.16	(− 0.95, 0.60)	0.85	(0.12, 1.11)
TG + P at 12 months	−0.59	(−0.83, − 0.02)	−0.23	(− 0.48, 0.14)	−0.45	(− 1.38, 0.17)	0.09	(− 0.41, 0.59)

CI confidence interval; OOP out-of-pocket; TG Treatment Group; TG + P Treatment Group plus Psychotherapy; UC Usual Care; eβ-1*100 = percent change in cost.

#### Health system costs for mental healthcare

**Fig. 5:**
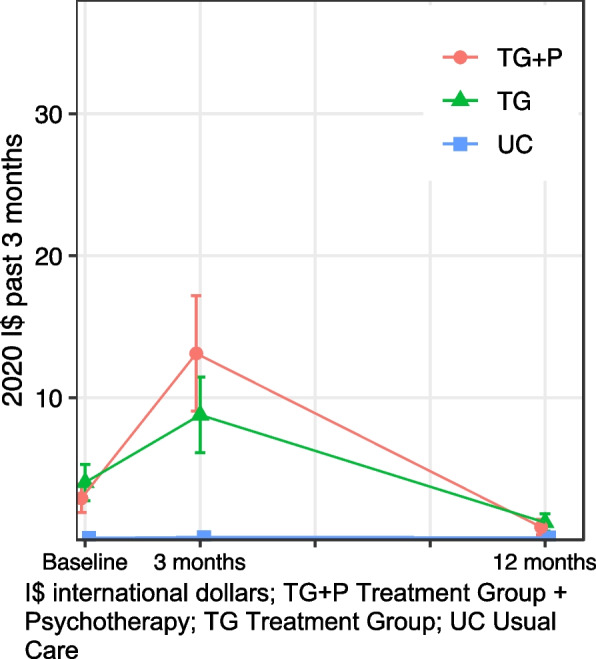
Mental healthcare costs

Trends in the societal costs of mental healthcare generally reflect those of all healthcare use, i.e., in an inverse “V” shape, among treatment goups (Fig. 5, Table 3). Healthcare costs were nonsignificantly lower at baseline among TG + P (difference = − 22, 95% CI: − 61, 11%), though there is significantly greater increase (difference = 85%; 95% CI: 12, 111%) in health system costs from baseline to 3 months among TG + P relative to TG. Health system costs of mental healthcare substantially decline for all three groups at 12-month follow up. Costs for mental healthcare remained negligible at all time points among the UC group given the low overall levels of mental healthcare use.

#### Individual out-of-pocket healthcare costs

**Fig. 6:**
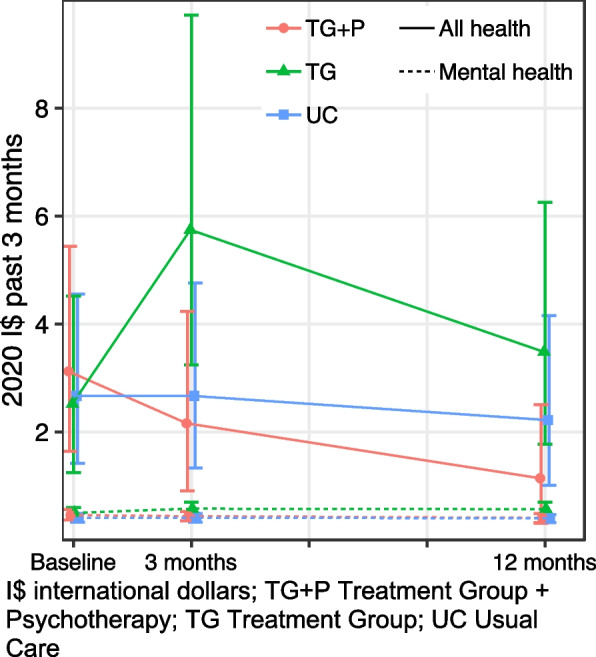
Individual healthcare expenditure. I$ international dollars; TG + P Treatment Group + Psychotherapy; TG Treatment Group; UC Usual Care

Trends of individual OOP healthcare costs differed than those of healthcare use (Table 3, Fig. 6). OOP costs were similar at baseline among the three groups: TG = 2.52 (95% CI: 1.25, 4.52), TG + *P* = 3.12 (95% CI: 1.64, 5.44), and UC = 2.67 (95% CI: 1.42, 4.56). Among TG, OOP costs increased 92% (95% CI: 6, 246%) and 27% (95% CI: − 30, 133%) at 3 and 12 months, respectively, relative to baseline values, though 12-month values were not significantly different (Table 3). In contrast, the difference in OOP expenditure among TG + P were 60% (95% CI: − 83, − 6%) and 59% (95% CI: − 83, − 2%) lower relative to TG differences at 3 and 12 months, respectively. Expenditure among UC remained relatively stable throughout the 12-month follow up: baseline = 2.67 (95% CI: 1.42, 4.56), 3 months = 2.66 (95% CI: 1.33, 4.76), and 12 months = 2.22 (95% CI: 1.01, 4.16).

We found very little OOP spending on mental healthcare overall and no differences between study groups, follow up, or study groups over time (Table 3, Fig. 6). This is likely because nearly all mental health services were provided by PRIME at no cost to the service user, though individuals may still incur expenses from transportation, antidepressant prescriptions not filled at PRIME facilities, or other cost drivers.

## Discussion

We examined outpatient healthcare use and costs over time among one group of individuals with depression who received a standard package of integrated mental health services, a second group of individuals with depression who received this package plus individual psychotherapy, and a third group of individuals with subclinical depressive symptoms who received care as usual. Healthcare use was low at the start of the study and increased from baseline to three months among all three groups, with increases in use generally corresponding to the level of mental health services provided by the PRIME project to each group (i.e., a high level of PRIME services among TG + P, a moderate level of PRIME services among TG, or no PRIME services among UC). Increases in healthcare use at the three-month follow up were largely driven by mental healthcare, though we also found greater healthcare use for physical health among the subclinical depressive symptoms group at three months. All categories of healthcare use returned to the low baseline levels among all three groups at the 12-month follow-up. Given short-term increases in use were driven by attending PRIME services, low use at long-term follow up likely reflects the discontinuation of mental healthcare use by individuals.

Healthcare use, driven by mental health service use, reflects expected trends and corresponds to the level of care provided under the PRIME group allocation. Depression services, including the 6–8 weekly session psychotherapy provided to the TG + P group, were primarily provided in the first three months of the study, which aligns with guidelines for care in the mhGAP Intervention Guide [59] and the observed increases in short-term use. Another explanation for observed increases in health visits could be that the additional visits were a byproduct of regular interactions with healthcare workers since healthcare workers encouraged service users to attend regular physical and mental health services during home follow-up as a part of their role. Low mental healthcare use at long-term follow up may be explained by individuals having completed structured interventions in the short-term, such as the Health Activity Program delivered to TG + P participants [60]; individuals discontinuing care after depressive symptoms improved [61]; or individuals needing fewer healthcare visits for treatment maintenance as recommended in the mhGAP Intervention Guide [59].

Healthcare use for physical health remained less than two visits per three months on average across all groups and time points. Short-term increases in use among the individuals with subclinical depression, i.e., the UC group, were driven by outpatient visits for physical health concerns. Physical healthcare visits among this group likely included help seeking for both physical and mental health conditions; visit type was categorized by self-reported presenting concerns and symptoms of mental disorders often present as physical ailments (e.g., headaches, body pains) among people with depression in Nepal [22, 23]. Conversely, physical health concerns among individuals in the two treatment groups may have been addressed during PRIME mental services, either by treating psychosomatic symptoms or health workers addressing physical health complaints during mental health service delivery.

Individual OOP costs were greatest among TG at 3-month follow up, despite similar OOP costs at baseline across the three groups. Results of post hoc analysis indicated increases in expenditure were due to differences in the location and type of healthcare being received. Those in the TG reported greater healthcare use for physical health as well as more frequently receiving care at traditional healers and specialists, both of which charged higher consultation fees on average than local health centers. Previous research from PRIME Nepal found mhGAP-based care to be less effective in reducing depressive symptoms and functional impairment when psychotherapy was not provided [31]. Combined with our findings, this evidence may indicate people who received less effective mental health services, i.e., TG, sought supplemental mental and physical healthcare elsewhere. PRIME services also included home-based follow up for both treatment groups, thus reducing OOP expenditure on transportation for mental healthcare and increasing contacts with the health system. We see a similar pattern when expanding our focus to include all health system costs: those receiving TG had greater health system costs at 3-month follow up, driven by higher OOP costs, despite lower overall healthcare use. Limiting health system costs to those for mental healthcare reflected expected trends; health system costs for mental health reflected the level of mental healthcare provided under PRIME Nepal.

To our knowledge, our study the is first to document differences in healthcare use and costs over time among individuals receiving different packages of integrated services for depression within primary care in LMIC. Total healthcare costs were greater among individuals diagnosed with depression compared to individuals without depression in the UC group. This finding aligns with previous cross-sectional research in Nepal [21] and other PRIME country sites [32] indicating households with at least one family member with depression have greater healthcare costs than households that do not, as well as with the well-documented economic burden of depression in high-income countries and LMIC [17–20].

Our findings should be interpreted alongside limitations. First, participants were nonrandomly assigned to UC, which is comprised of those who screened positive for depression but were not diagnosed during clinical interview. There were no differences in depressive symptoms at baseline between treatment and UC groups, though functional impairment was lower at baseline among UC individuals [31]. This difference in functional impairment between those with depression and without depression is expected: those with subclinical symptoms experienced lower impairment. Increases in physical healthcare use at follow up may also reflect a greater physical disease burden among UC participants, though physical healthcare use at baseline was similar among all groups. Though imperfect, the UC group provides the closest available comparator of service users experiencing depressive symptoms within the primary healthcare system in Nepal. As such, we have included UC in the present study as an imperfect comparator and intentionally avoided referring to this group as a control. Second, we examined the costs of *delivering* health services according to self-reported resource use and published unit costs for different providers, locations, and medications. Costs associated with training and implementing various mental and physical health services utilized by service users, including those of PRIME Nepal, are beyond the scope of this paper. Future research should investigate whether integrated mental health services provided by PRIME represent cost-effective investments when considering both training and implementation costs. Our findings on the economic impact of integrated services to the service user and health system are limited in scope to service delivery alone. This is true of both PRIME services and other services utilized by study participants, i.e., implementation costs for physical health services are also excluded from our analyses. Lastly, psychotropic medications costs were estimated and assigned to primary care facilities under health systems costs, rather than directly reported by service users or healthcare administrators. Our measure of healthcare use, the CSRI, did not specifically ask respondents to report expenditure on psychotropic medications, though these costs may have been indirectly reported by service users as consultation fees. Since most, if not all, psychotropic medications were provided at no cost to the service users by PRIME health facilities, our approach is likely a close approximation of actual payers and costs.

## Conclusion

Two packages of integrated mental health services for depression did not differ in costs to the health system for delivery or service user for utilization despite one of these packages also including individual psychotherapy, which has been found to be the service component that determined effectiveness for treating depression in this context. So, adding psychotherapy to mental health services within primary care significantly increases and determines the effectiveness of depression care, at no additional costs to the health system for service delivery. This is a highly policy-relevant finding, as the combination of knowing what is needed in terms of services to reduce depression and knowing this does not result in undue financial burden to the individual or health system provides for the key arguments for scaling up such services. While our findings provide encouraging results for service users, further research is needed incorporating both implementation and service delivery costs to estimate the full value of integrated mental healthcare to the health system. Future research is also needed on the impact of improved mental health on physical healthcare use; though physical healthcare use was slightly lower among treatment groups compared to usual care over follow up, we were unable to make conclusive statements in this area given the low overall levels of physical healthcare use observed and our sample size. Lastly, we provide evidence individuals often rely on mental and physical health services outside the formal healthcare system, highlighting the continuing role of informal healthcare providers in Nepal. Our findings taken together can be used to inform how, and at what cost, integrated mental health services for depression can be delivered within primary care in Nepal and similar contexts.

## Supplementary Information

**Additional file 1:** **Supplementary Fig. S1.**

## Data Availability

The data supporting the findings is available through the PRIME program’s website: http://www.prime.uct.ac.za/.

## Abbreviations

CI: confidence interval
CSRI: Client Socio-Demographic and Service Receipt Inventory
I$: international dollars
IR: incidence rate
IRR: incidence rate ratio
mhGAP: Mental Health Gap Action Programme
OOP: out-of-pocket
PHQ-9: Patient Health Questionnaire, 9-item version
PRIME: Programme for Improving Mental Health Care
SD: standard deviation
TG: Treatment Group
TG + P: Treatment Group plus Psychotherapy
UC: Usual Care
WHO: World Health Organization.
