# Supplementary material for: Healthcare use and costs among individuals receiving mental health services for depression within primary care in Nepal
Source: BMC Health Serv Res. 2022 Dec 30;22:1596. doi: 10.1186/s12913-022-08969-1 (PMC9804956; doi:10.1186/s12913-022-08969-1)
Supplement: Supplementary file 1 — Additional file 1: Supplementary Fig. S1. [file 12913_2022_8969_MOESM1_ESM.docx]

**SUPPLEMENT**

**Figure S1.** CONSORT Flow Diagram

Assessed for eligibility (n=2044)

Excluded (n=1852)

– Received no diagnosis (n=1630)

– Received other diagnosis (n=205)

– Did not consent (n=17)

Analysed (n=59)

Excluded from analysis (n=1)

–  Outlier (n=1)

Lost to follow-up (n=9)

– Moved away (n=7)

– Refused (n=2)

Allocated to TG (n=60)

– Received allocated intervention (n=60)

Lost to follow-up (n=14)

–  Moved away (n=6)

– Refused (n=6)

– Kept cancelling (n=1)

–  Hospitalized (n=1)

Allocated to TG+P (n=60)

– Received allocated TG+P (n=52)

– Did not receive allocated TG+P (n=8)

Analysed (n=60)
Excluded from analysis (n=0)

**Enrollment**

Randomized (n=120)

Diagnosed depression

No intervention allocated (n=71); received usual care

Lost to follow-up (n=4)

– Moved away (n=3)

– Refused (n=1)

Analyzed (n=69)

Excluded from analysis (n=2)

–  Outliers (n=2)

Nonrandomized (n=71)

Subclinical symptoms

**Follow-Up**

**Allocation**

**Analysis**

Treatment Group (TG, n=60)

Treatment Group plus Psychotherapy (TG+P, n=60)

Usual Care (n=71)
